# Posterior Splenic Abscess Leak Causing Salmonella Group B Peritonitis in an Immunocompetent Young Adult

**DOI:** 10.7759/cureus.101103

**Published:** 2026-01-08

**Authors:** Michael W Alchaer, Paul Farag, Insoon Park, Thomas A Abbruzzese

**Affiliations:** 1 General Surgery, HCA Healthcare/University of South Florida (USF) Morsani College of Medicine Graduate Medical Education (GME) HCA Florida Brandon Hospital, Brandon, USA

**Keywords:** immunocompetent host, laparoscopic management, peritonitis, salmonella group b, splenic abscess

## Abstract

Splenic abscess is an uncommon intra-abdominal abscess, typically associated with immunocompromised states. This report highlights a case where the abscess was caused by Salmonella species in an immunocompetent individual, which is a very rare occurrence with limited prior documentation.

We report a previously healthy 24-year-old male who presented with high-grade fever and diffuse abdominal pain. Preoperative CT imaging demonstrated hepatosplenomegaly and a 5.6 × 3.8 cm posterior splenic lesion. Exploratory laparoscopy revealed four-quadrant purulent peritonitis without hollow-viscus perforation and a posterior splenic capsular tear with oozing. Cultures from blood, peritoneal fluid, and a subsequent percutaneous splenic drain all grew Salmonella enterica serogroup B.

Spontaneous leak of a splenic abscess causing diffuse peritonitis without gastrointestinal perforation is extremely rare. While most reported cases involve comorbidities, Salmonella splenic abscesses have been documented in healthy individuals. Management requires prompt source control, either percutaneous drainage or splenectomy, plus targeted antimicrobials. Our patient was successfully treated with laparoscopic washout, targeted antibiotic therapy after allergy-related adjustment, and image-guided drainage, preserving splenic function.

This case highlights one of the few documented instances of Salmonella Group B splenic abscess rupture in an immunocompetent adult. Clinicians should maintain high suspicion for splenic pathology in Salmonella bacteremia with peritonitis, even without classic risk factors. Organ-sparing approaches can be effective when combined with multidisciplinary care.

## Introduction

Splenic abscesses are uncommon, representing less than 1% of all intra-abdominal abscesses [1,2]. They typically occur in patients with underlying immunocompromised conditions such as diabetes, end-stage renal disease, or malignancy [3]. Salmonella species are an unusual etiology and are most often reported in individuals with significant comorbidities [4,5]. Splenic abscesses most commonly arise from hematogenous seeding during bacteremia, with Staphylococcus, Streptococcus, and Escherichia coli among the most frequently isolated organisms [1-5].

Spontaneous rupture of a splenic abscess leading to diffuse peritonitis is exceptionally rare and can clinically mimic hollow-viscus perforation [6-8]. Although Salmonella peritonitis more commonly arises from gastrointestinal perforation or peritoneal dialysis [9,10], cases of splenic abscess rupture in otherwise healthy, immunocompetent adults have been documented [11,12].

## Case presentation

A 24-year-old male with no significant past medical or surgical history presented with a two-day history of high-grade fever (maximum temperature 40.5°C) and progressively worsening diffuse abdominal pain involving all four quadrants. The pain was constant, severe, and associated with generalized abdominal tenderness. He denied nausea, vomiting, diarrhea, hematochezia, or recent abdominal trauma.

On physical examination, the patient was febrile and tachycardic, with diffuse abdominal tenderness and guarding. Initial laboratory evaluation demonstrated stable hemoglobin levels, no leukocytosis, and an otherwise unremarkable metabolic panel. Blood cultures obtained on preoperative day 1 later grew Salmonella enterica serogroup B.

**Figure 1:**
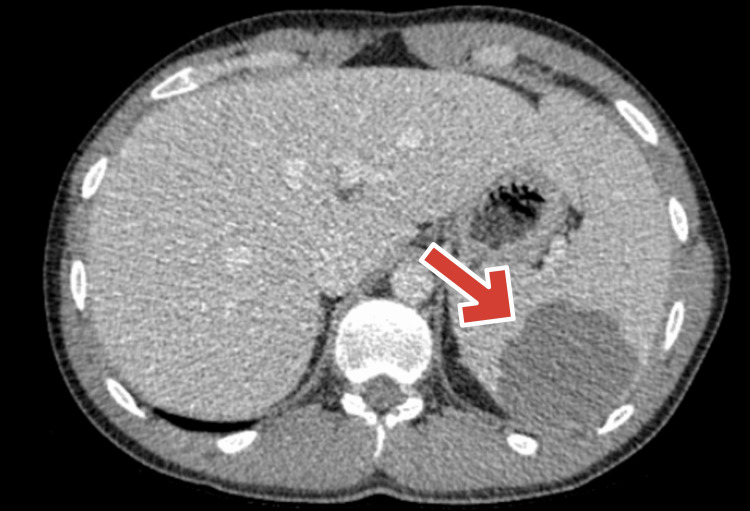
Contrast-enhanced CT abdomen/pelvis (pre-op day 1) showing a 5.6x3.8 cm posterior splenic lesion with hepatosplenomegaly. Arrow pointing at the splenic lesion.

Contrast-enhanced CT of the abdomen and pelvis on pre-op day one demonstrated hepatosplenomegaly and a 5.6x3.8 cm posterior splenic lesion (Figure 1).

Empiric broad-spectrum intravenous antibiotics were initiated preoperatively upon suspicion of intra-abdominal infection. On pre-op day 0, worsening abdominal pain and rebound tenderness prompted exploratory laparoscopy. Intraoperative findings included four-quadrant purulent peritonitis without evidence of hollow viscus perforation. A posterior splenic capsular tear with localized oozing was identified; the tear was associated with localized oozing without evidence of fibrosis, scarring, or chronic inflammatory changes, suggesting a recent capsular disruption likely related to abscess expansion rather than prior trauma. A 19 Fr Jackson-Pratt (JP) drain was placed in the left upper quadrant adjacent to the posterior splenic capsule. Peritoneal fluid cultures obtained intraoperatively grew Salmonella Group B.

The patient was started on intravenous ceftriaxone; however, he developed an allergic rash on post-op day two, prompting a switch to meropenem. He remained hemodynamically stable but continued to have localized abdominal tenderness.

**Figure 2:**
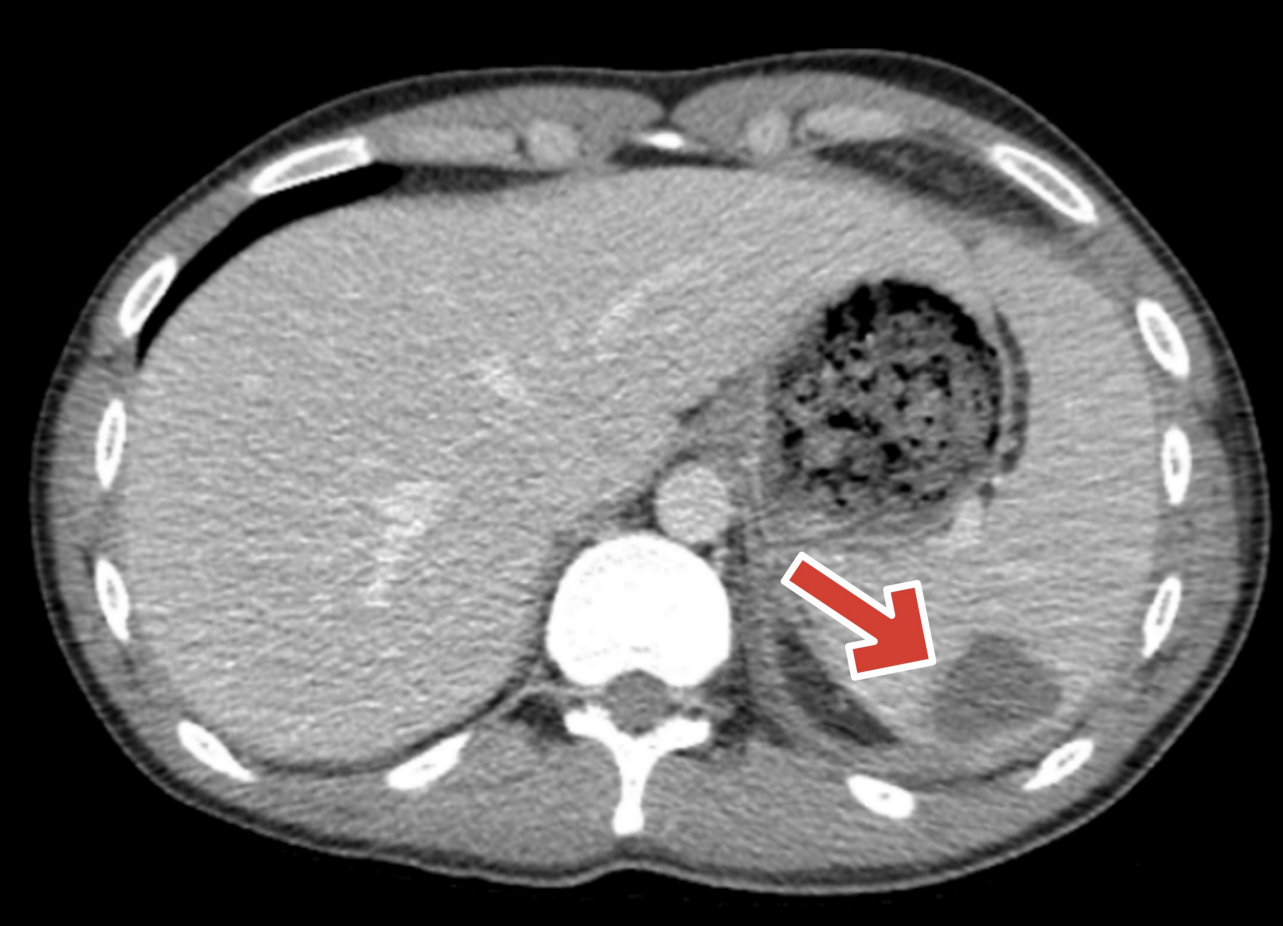
Post-op day 18 CT abdomen/pelvis showing interval decrease in size of splenic collection after drainage (2.2x1.8cm). Arrow pointing at the splenic collection.

On post-op day 13, interventional radiology placed a percutaneous drain into the posterior splenic lesion. Culture from this drain also grew Salmonella Group B, confirming the spleen as the source of infection. All other cultures were negative. A CT scan on post-op day 18 demonstrated decreased size of the splenic collection (2.2x1.8 cm) and improved associated inflammation (Figure 2). All drains were removed by post-op day 20, and the patient was transitioned to oral trimethoprim-sulfamethoxazole for completion of therapy. He was discharged in stable condition without complications.

## Discussion

Splenic abscess is a rare clinical entity, accounting for less than 1% of intra-abdominal abscesses and typically affecting individuals with underlying immunocompromised states such as diabetes, end-stage renal disease, or malignancy [1-3]. Salmonella species represent an uncommon cause, most frequently occurring in patients with comorbidities; however, isolated reports describe splenic abscesses in otherwise healthy adults [4,5,11,12].

Spontaneous rupture or leak of a splenic abscess leading to diffuse peritonitis without gastrointestinal perforation is exceptionally uncommon. Published case reports and small series emphasize the importance of considering splenic pathology when diffuse peritonitis occurs in the absence of bowel perforation [6-8,13]. In the present case, the diagnosis was confirmed microbiologically, as Salmonella Group B was isolated from blood, peritoneal fluid, and percutaneous splenic drainage cultures, confirming the spleen as the infection source [4,12,14].

While most cases described in the literature involve patients with significant comorbidities or immunosuppression, splenic abscesses secondary to Salmonella infection have been reported in immunocompetent individuals [11,12,15]. In such patients, splenic preservation is feasible when adequate source control is achieved through image-guided drainage or minimally invasive washout, supplemented with targeted antimicrobial therapy. This spleen-sparing approach mitigates the lifelong risk of postsplenectomy sepsis while maintaining immune function.

Management strategies for splenic abscesses depend on abscess size, number, and patient stability. Standard treatment involves prompt source control through either percutaneous drainage or splenectomy, coupled with pathogen-directed antibiotics and follow-up imaging to ensure resolution [4,5,15]. Although Salmonella peritonitis more commonly results from gastrointestinal perforation or peritoneal dialysis [9,10,16,17], clinicians should maintain vigilance for splenic involvement when Salmonella bacteremia presents with diffuse peritoneal signs.

## Conclusions

This case highlights an unusual presentation of Salmonella Group B peritonitis originating from a posterior splenic abscess leak in an immunocompetent young adult, one of the very few such cases documented in the literature. Prompt recognition, early operative washout, targeted antibiotic therapy after allergy-related regimen adjustment, and image-guided drainage achieved effective source control while preserving splenic function.
